# Salvage Radiotherapy Confers an Overall Survival Advantage in Biochemical Recurrence of Prostate Cancer: Evidence from the International PROMISE Registry

**DOI:** 10.2967/jnumed.125.271770

**Published:** 2026-08

**Authors:** Nika Guberina, Madeleine J. Karpinski, Maja Guberina, Sebastian Hoberück, Matthias Miederer, Tobias Hölscher, Isabel Rauscher, Matthias Eiber, Andrew Nguyen, Lela Theus, Matteo Bauckneht, Helen Scholtissek, Kambiz Rahbar, Jonathan Miksch, Daniele Antonio Pizzuto, Karolien Goffin, Caner Civan, Shima F. Reinwand, Andreas Stang, Jens Kleesiek, Anja Merkel-Jens, Lale Umutlu, Alfonso Santangelo, Timo F. W. Soeterik, Stephan Beintner-Skawran, Andrej Vondrak, Anders Bjartell, Laura Evangelista, Sazan Rasul, Marcin Miszczyk, Adrien Holzgreve, Gabriel T. Sheikh, Andrea Di Giorgio, Andrea Farolfi, Ken Herrmann, Boris Hadaschik, Wolfgang P. Fendler, Martin Stuschke

**Affiliations:** 1 Department of Radiotherapy, West German Cancer Center, NCT, University Hospital Essen, Essen, Germany;; 2 DKTK, Partner Site University Hospital Essen, Germany;; 3 Department of Nuclear Medicine, DKTK and NCT University Hospital Essen, Essen, Germany;; 4 Department of Nuclear Medicine, University Hospital Münster and West German Cancer Center, Münster, Germany;; 5 Department of Nuclear Medicine, University Hospital Carl Gustav Carus, Technical University Dresden, Dresden, Germany;; 6 Department of Translational Imaging in Oncology, NCT/UCC Dresden: Faculty of Medicine and University Hospital Carl Gustav Carus, TUD, Dresden, Germany;; 7 Department of Radiotherapy and Radiation Oncology, Faculty of Medicine, University Hospital Carl Gustav Carus, TUD Dresden University of Technology, Dresden, Germany and;; 8 Department of Nuclear Medicine, School of Medicine and Health, TUM University Hospital, Munich, Germany;; 9 Bavarian Center for Cancer Research, Erlangen, Germany;; 10 Ahmanson Translational Theranostics Division, Department of Nuclear Medicine and Theranostics, David Geffen School of Medicine at UCLA, Los Angeles, California;; 11 IRCCS Ospedale Policlinico San Martino, Genoa, Italy;; 12 Department of Health Sciences, University of Genoa, Genoa, Italy;; 13 Nuclear Medicine, Faculty of Medicine, University of Augsburg, Augsburg, Germany;; 14 Department of Radiology, Ulm University Hospital, Ulm, Germany;; 15 Department of Nuclear Medicine, Ulm University Hospital, Ulm, Germany;; 16 Direzione Scientifica GSTeP Radiopharmacy, Department of Radiology and Radiotherapy, Fondazione Policlinico Universitario A. Gemelli IRCCS, Rome, Italy;; 17 Nuclear Medicine, University Hospitals Leuven, and Nuclear Medicine & Molecular Imaging, Department of Imaging and Pathology, KU Leuven, Leuven, Belgium;; 18 Institute of Medical Informatics, Biometry and Epidemiology, University Hospital Essen, Essen, Germany;; 19 Cancer Registry North-Rhine Westphalia gGmbH, Bochum, Germany, and University Hospital Essen, Essen Germany;; 20 Institute for AI in Medicine, University Medicine Essen, Essen, Germany;; 21 Department of Diagnostic and Interventional Radiology and Neuroradiology, University Hospital Essen, Essen, Germany;; 22 Unit of Urology, Division of Oncology, Gianfranco Soldera Prostate Cancer Lab, IRCCS San Raffaele Scientific Institute, VitaSalute San Raffaele University, Milan, Italy;; 23 Department of Radiation Oncology, University Medical Center Utrecht, Utrecht, The Netherlands;; 24 Department of Urology, St. Antonius Hospital Utrecht, Utrecht, The Netherlands;; 25 Department of Nuclear Medicine, University Hospital Zurich, University of Zurich, Zurich, Switzerland;; 26 IZOTOPCENTRUM, s.r.o, Nuclear Medicine Department, Nitra, Slovakia;; 27 Department of Urology, Skåne University Hospital, Malmö, Sweden;; 28 Department of Translational Medicine, Lund University, Malmö, Sweden;; 29 Department of Biomedical Sciences, Humanitas University, Pieve Emanuele, Milan, Italy;; 30 Nuclear Medicine Unit, IRCCS Humanitas Research Hospital, Milan, Italy;; 31 Division of Nuclear Medicine, Department of Biomedical Imaging and Image-Guided Therapy, Medical University of Vienna, Vienna, Austria;; 32 Department of Urology, Comprehensive Cancer Center, Medical University of Vienna, Vienna, Austria;; 33 Collegium Medicum, Faculty of Medicine, WSB University, Dąbrowa Górnicza, Poland;; 34 Department of Nuclear Medicine, LMU University Hospital, LMU Munich, Munich, Germany;; 35 Nuclear Medicine, Alma Mater Studiorum, University of Bologna, Bologna, Italy;; 36 Nuclear Medicine, IRCCS Azienda Ospedaliero-Universitaria Di Bologna, Bologna, Italy; and; 37 Department of Urology, University Hospital Essen, Essen, Germany

**Keywords:** biochemical recurrence, molecular imaging, overall survival, prostate cancer, salvage radiotherapy

## Abstract

The impact of salvage radiotherapy (SRT) on overall survival (OS) in patients experiencing biochemical recurrence (BCR) of prostate cancer remains an area of active investigation. The international multicenter PROMISE registry provides a valuable dataset to explore the association between SRT and long-term clinical outcomes in this patient population (NCT06320223). **Methods:** Comprehensive restaging at the time of BCR was performed by integrating both serologic markers and advanced molecular imaging (PET/CT or PET/MRI), offering an accurate assessment of molecular imaging (mi) TNM stage. **Results:** In total, 1410 patients experiencing BCR with prior prostatectomy were included. The median follow-up was 5.2 y (interquartile range, 3.8–6.9 y). SRT was administered after PET to 381 of 680 patients (56.0%) with PET-negative disease (miT0N0M0) and to 478 of 730 patients (65.0%) with PET-locoregional disease (miT/N+ M0). SRT was associated with longer OS in the entire cohort (hazard ratio, 0.66; 95% CI, 0.47–0.93; *P* = 0.019) with 5- and 7-y survival rates of 95.2% (95% CI, 93.6%–96.8%) and 90.8% (95% CI, 88.1%–93.6%), respectively. Without SRT, 5- and 7-y survival rates were 92.1% (95% CI, 89.6%–94.7%) and 83.8% (95% CI, 79.8%–88.1%), respectively. Subgroup analysis found significant benefit for patients with miT0N0M0 disease (hazard ratio, 0.42; 95% CI, 0.22–0.78; *P* = 0.0061), particularly those with a prostate-specific antigen level of 0.5 ng/mL or lower (*P* = 0.03, log-rank test). **Conclusion:** SRT improves OS for patients with BCR, especially those with a negative prostate-specific membrane antigen–targeted PET.

## Visual Abstract

**Figure:**
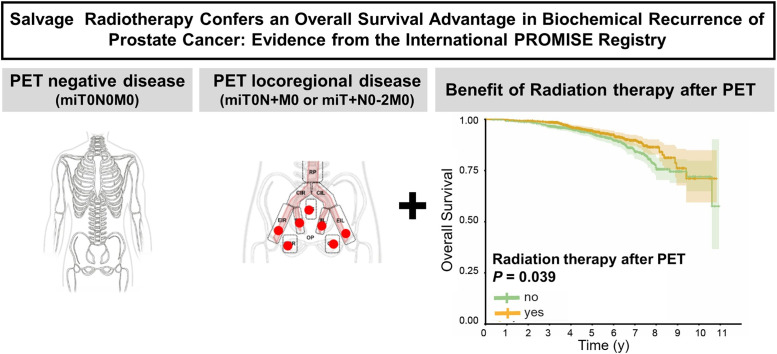

Primary curative-intent therapy options for clinically significant prostate cancer include radical prostatectomy and definitive radiotherapy (1,2). Both therapeutic modalities are highly efficacious, with excellent control rates for most men with locally confined disease (3–5). However, depending on the initial extent of the tumor and tumor-specific risk factors, patients may suffer from biochemical relapse. After radical prostatectomy, salvage radiotherapy (SRT) is an accepted therapy option in the case of biochemical recurrence (BCR) (1). Several studies effectively demonstrated that SRT and early SRT have a comparable effect, but a difference in survival with or without SRT has not been shown (6–9). National and international guidelines state that negative imaging at the time of recurrence should not delay the initiation of SRT with curative intent (10).

In previous studies, the biochemical progression-free and overall survival (OS) of patients undergoing SRT was primarily reported for subgroups with various preoperative and postoperative risk factors, rather than a restaging group at BCR with prostate-specific membrane antigen (PSMA) PET/CT. Holzgreve et al. found positive PSMA PET results for 84% of patients with high-risk BCR after prostatectomy with no metastatic disease on conventional imaging and detected M1 disease in 46% of patients (11). Thus, PET results can impact decision-making for SRT or can change target volumes (12).

This study utilized data from a large multicenter registry with detailed documentation of PSMA PET–based restaging to investigate the association between SRT and OS after prostatectomy in patients without evidence of distant metastases.

## MATERIALS AND METHODS

### Study Design

This cohort study involved patients from the ongoing PROMISE registry (NCT06320223; http://promise-pet.org) with histologically proven prostate cancer recruited from 20 sites worldwide. The ethics committee of the University Hospital Essen approved the present study (23-11501-BO), and the data were centrally collected in a REDCap database. For survival analysis and examination of potential predictive value of radiotherapy, 2 patient groups were differentiated by whether or not radiotherapy was used as the primary treatment after PET restaging.

### Participants

Patients were included if they underwent PSMA PET to assess BCR or prostate-specific antigen (PSA) persistence after radical prostatectomy, had known PSA level, and had a management plan after PET. BCR after radical prostatectomy was defined as a PSA level that did not decrease to undetectable levels (PSA persistence), an undetectable PSA level with a subsequent detectable PSA level that increased on at least 2 determinations (BCR), or an increase of more than 0.1 ng/mL in PSA level (1). Patients were excluded if they had missing OS follow-up data, a neuroendocrine pattern at diagnosis, or distant metastases (miM+) on PSMA PET.

On the basis of PSMA PET restaging, patients were classified as having PET-negative disease (miT0N0M0) or PET-locoregional disease (miT0 N+ M0 or miT+ N0–2 M0; expressed as miT/N+ M0 from this point forward). Patients were divided into subgroups on the basis of whether or not they received SRT after PET.

### Procedures, Outcomes, and Statistical Analysis

PSMA PET imaging was performed in accordance with international consensus guidelines and analyzed by experienced radiologists and nuclear medicine physicians. Patients underwent PSMA PET/CT or whole-body PET/MRI using [^68^Ga]Ga-PSMA-11, [^18^F]F-PSMA-1007, or other PSMA tracers. Prostate cancer spread was described in accordance with PROMISE version 2 criteria, and miTNM was documented (13). OS was the primary outcome, defined as the time from PSMA PET to death from any cause. Kaplan–Meier survival curves and respective log-rank tests were used, and hazard ratios were calculated using Cox proportional analyses. This is an ongoing and growing registry study. Prespecified sample size calculations were not performed.

Survival curves for patients with PET-negative disease were constructed for patients with any PSA level at the time of PSMA PET and for patients with PSA values of at least 0.5 ng/mL. This cutoff value was chosen to build homogeneous BCR groups, allowing for enhanced risk stratification and better effect interpretation.

Survival curves for all patients who underwent either radical prostatectomy or definitive radiotherapy before PSMA PET are shown in Supplemental Figure 1, available at http://jnm.snmjournals.org.

All statistical analyses were performed with R version 4.2.1.

## RESULTS

**FIGURE 1.**
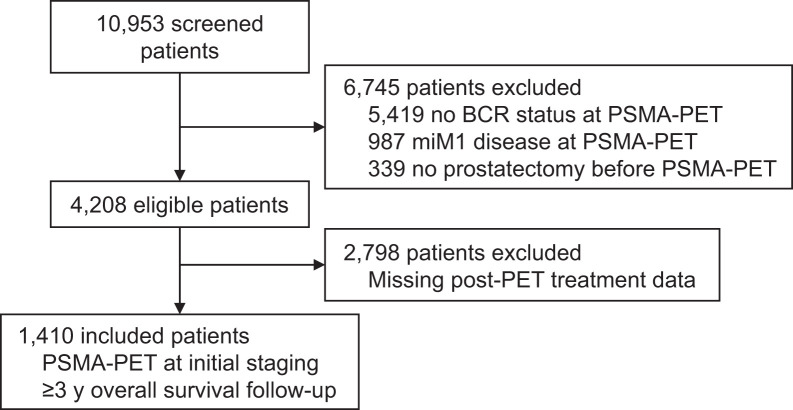
Flowchart of screened patients from PROMISE registry.

At the time of data cutoff, 10,953 patients with prostate cancer were enrolled in the PROMISE registry, 4547 of whom underwent PSMA PET for restaging purposes. Of those, 2798 were excluded because of a missing management plan after PET. Overall, 1410 patients (80.6%) with BCR who underwent radical prostatectomy before PSMA PET between May 13, 2013, and October 27, 2024, were included in the study (Fig. 1).

Patient characteristics are presented in Table 1. The median age and management plan after PET were similar between groups, but PSA levels differed. In total, 393 patients (57.8%) with PET-negative disease and 209 patients (28.6%) with PET-locoregional disease had PSA levels of less than 0.5 ng/mL. Most patients underwent PSMA PET with a [^68^Ga]Ga-PSMA-11, followed by [^18^F]F-PSMA-1007 or another PSMA tracer. At the time of data cutoff, 44 deaths (6.5%) from any cause had occurred among patients with PET-negative disease, with a median follow-up of 5.2 y (interquartile range [IQR], 3.6–6.8 y). In comparison, 86 deaths (11.8%) from any cause had occurred among patients with PET-locoregional disease, with a median follow-up of 5.2 y (IQR, 3.9–7.0 y).

**TABLE 1.** Patient Characteristics

Characteristic	PET-negative disease (miT0 N0 M0)	PET-locoregional disease (miT/N+ M0)	Total cohort
*n*	680 (48.2)	730 (51.8)	1410 (100)
Age at PET (y)	69 (63–74)	70 (65–75)	70 (64–74)
PSA level at PET (ng/mL)			
<0.5	393 (57.8)	209 (28.6)	602 (42.7)
≥0.5	287 (42.2)	521 (71.4)	808 (57.3)
Management after PET			
Radiation therapy	381 (56.0)	478 (65.5)	859 (60.9)
ADT	105 (35.1)	107 (42.5)	212 (38.5)
ARPI	13 (4.3)	7 (2.8)	20 (3.6)
Chemotherapy	NA	3 (1.2)	3 (0.5)
Other*	181 (60.3)	135 (53.6)	237 (57.2)
PSMA PET tracer			
[^68^Ga]Ga-PSMA-11	601 (88.4)	552 (75.6)	1153 (81.8)
[^18^F]PSMA-1007	53 (7.8)	73 (10)	126 (8.9)
Other tracer	23 (3.4)	104 (14.2)	127 (9.0)
Missing	3 (0.4)	1 (0.1)	4 (0.3)

* Includes immunotherapy, combination therapies, watchful waiting, active surveillance, or no therapy.

ADT = androgen-deprivation therapy; ARPI = androgen receptor pathway inhibitor.

Qualitative data are expressed as number and percentage; continuous data are expressed as median and IQR.

SRT was administered after PET to 381 (56.0%) of 680 patients with miT0N0M0 disease and 478 (65.0%) of 730 patients with miT/N+ M0 disease. Prostate cancer spread by miTNM and total number of lesions by radiotherapy status are presented in Table 2. The median time from PET to SRT was 2.2 mo (IQR, 1.0–5.3 mo) for patients with miT0N0M0 disease and 1.8 mo (IQR, 1.0–2.8 mo) for those with miT/N+ M0 disease.

**TABLE 2.** PSMA PET Restaging Findings Before Treatment

	No RT after PET			RT after PET		
Variable	miT0 N0 M0	miT/N+ M0	Total	miT0 N0 M0	miT/N+ M0	Total
miT						
miT0	299 (100)	125 (49.6)	424 (77.0)	381 (100)	241 (50.4)	622 (72.4)
miT2		5 (2.0)	5 (0.9)		2 (0.4)	2 (0.2)
miT3		1 (0.4)	1 (0.2)		1 (0.2)	1 (0.1)
miTr		121 (48.0)	121 (22.0)		234 (49.0)	234 (27.2)
miN						
miN0	299 (100)	97 (38.5)	396 (71.9)	381 (100)	187 (39.1)	568 (66.1)
miN1		112 (44.4)	112 (20.3)		215 (45.0)	215 (25.0)
miN2		43 (17.1)	43 (7.8)		76 (15.9)	76 (8.8)
Total lesion count						
0–5	299 (100)	210 (83.3)	509 (92.4)	381 (100)	404 (84.5)	785 (91.4)
6–20		2 (0.8)	2 (0.4)		4 (0.8)	4 (0.5)
NA		40 (15.9)	40 (7.3)		70 (14.6)	70 (8.1)
TTV	0	1.06 (0.24–3.22)	0 (0–0.51)	0	0.95 (0.13–2.3)	0 (0–0.95)

RT = radiation therapy; NA = not available; TTV = total tumor volume.

Qualitative data are expressed as number and percentage; continuous data are expressed as median and IQR.

**FIGURE 2.**
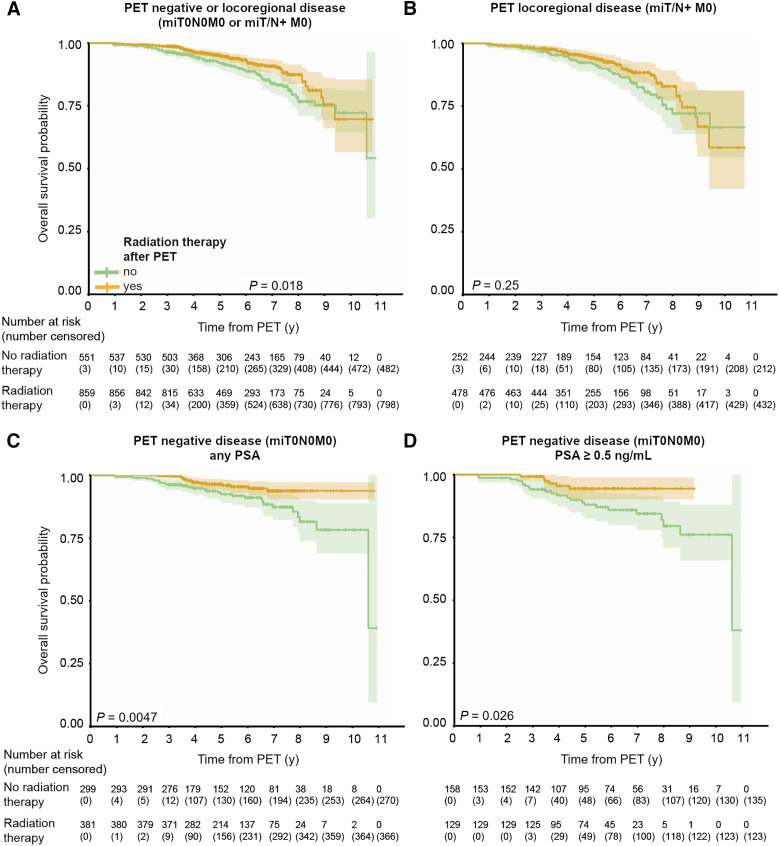
OS curves for all patients who underwent prostatectomy before PET with (A) PET-negative or PET-locoregional disease (miT0 N+ M0 or miT+ N0–2 M0), (B) PET-locoregional disease (miT/N+ M0), or PET-negative disease (miT0N0M0) at any PSA level (C) or PSA levels ≥0.5 ng/mL (D) at BCR differentiated by SRT status after PET.

In the total cohort, 5- and 7-y OS probabilities of 95.2% (95% CI, 93.6%–96.8%) and 90.8% (95% CI, 88.1%–93.6%), respectively, were observed for patients who received SRT after PET versus 92.1% (95% CI, 89.6%–94.7%) and 83.8% (95% CI, 79.8%–88.1%), respectively, in patients who did not receive SRT after PET (*P* = 0.02, log-rank test) (Fig. 2A). Survival curves are presented for patients with PET-locoregional disease (*P* = 0.25, log-rank test; Fig. 2B) and those with PET-negative disease with any PSA value (*P* = 0.005, log-rank test; Fig. 2C) and with PSA levels of at least 0.5 ng/mL (*P* = 0.026, log-rank test; Fig. 2D). The hazard ratios for patients who received SRT after PET were 0.66 (95% CI, 0.47–0.93; *P* = 0.02) for the entire cohort, 0.78 (95% CI, 0.51–1.19; *P* = 0.25) for patients with PET-locoregional disease, 0.42 (95% CI, 0.22–0.78; *P* = 0.006) for patients with PET-negative disease, 0.68 (95% CI, 0.63–0.73; *P* = 0.46) for patients with PET-negative disease and a PSA level of less than 0.5 ng/mL, and 0.37 (95% CI, 0.36–0.42; *P* = 0.033) for patients with PET-negative disease and a PSA level of at least 0.5 ng/mL.

Similar findings were observed for patients who received either radical prostatectomy or definitive radiotherapy before PSMA PET (Supplemental Fig. 1).

## DISCUSSION

The results of this study are based on the large multicenter PROMISE registry (NCT06320223), including patients with BCR who underwent PSMA PET regardless of PSA level. The median follow-up was longer than 5 y; therefore, OS was used as a robust primary endpoint. Other endpoints, such as event-free survival, where BCR is the most frequent event, did not consistently meet the criteria of a surrogate endpoint for OS after primary radiotherapy or SRT for prostate cancer in previous studies (14–16). At the survival endpoint, the proportion of cancer-related deaths critically depends on the risk profile of patients with BCR after prostatectomy and the follow-up time. Randomized trials of SRT in patients with a higher risk profile, as defined by CAPRA-S score, showed a crude mortality rate of 13.85% and a cancer-specific mortality rate of 5.25% (median follow-up, 8.9 y) (17), increasing to 28.45% and 7.98%, respectively, at a median follow-up of 13 y (18). In comparison, trials of adjuvant radiotherapy or SRT including patients with lower risk profiles showed cancer-specific and overall mortality rates of 1.51%–1.89% and 5.38%–5.66%, respectively, at median follow-up times of 5.2–6.2 y (7,19). Mogensen et al. recently showed the positive effect of PSMA PET on outcomes after SRT (20). The present analysis of the PROMISE registry is the attempt to provide greater insight into the effects of SRT on survival in molecular imaging subgroups at the time of BCR. SRT was significantly associated with improved OS across the entire cohort, with the largest effect seen patients with PET-negative disease. This underlines the importance of salvage radiation therapy in PET-negative disease at BCR. A significant association of SRT and OS could not be confirmed in patients with PET-locoregional disease. The lower therapy effect in this group can be partly attributed to advanced disease. On the other hand, the registry cohort was more heterogeneous than the PET-negative group; therefore, a clear statistical benefit could not be demonstrated.

A slightly higher proportion of patients receiving SRT had locoregional positive disease versus PET-negative disease. The decision to administer SRT to patients with BCR in the setting of miT0N0M0 was shared by the patient and treating physician. This is consistent with the finding that miT0N0M0 disease identified by PSMA PET/CT led to major treatment changes by the physician toward surveillance, whereas a locoregional disease finding led to SRT (21).

Previous trials, as the randomized EMPIRE-1 trial effectively demonstrated, have found that the inclusion of pre-SRT PET imaging into the SRT treatment plan significantly improved BCR rates or event-free survival, the latter including BCR rates (22). In the ^18^F-fluciclovine PET/CT arm of the latter trial, prostate bed irradiation was performed only in patients with no or only local tracer uptake, pelvic lymph node irradiation and prostate bed irradiation was performed in those with pelvic nodal uptake without extrapelvic uptake, and no SRT was administered to those with extrapelvic or skeletal uptake. PSMA PET radiopharmaceuticals can detect micrometastatic disease with a higher sensitivity and specificity compared with CT, MRI, and bone scan at both initial staging and at the time of BCR (1,22,23). The role of oligometastases-directed therapy in PSMA PET miM+ with distant metastases outside the pelvis after BCR is under current investigation, and data from randomized studies are emerging (24).

There is an indication for SRT in patients without distant metastases at BCR using conventional or molecular imaging, in accordance with interdisciplinary guidelines (1,2). This includes patient groups whose disease is defined as miT0N0M0 or miT/N+ M0. Although the risk of BCR is substantially reduced, randomized trials have not found a clear survival benefit with adjuvant radiotherapy or SRT versus no radiotherapy (12,25–28). Explanations for this may include the low number of events during the limited follow-up time and the large number of geographic misses after conventional restaging at the time of BCR. Thus, PSMA PET–positive lesions were found outside the prostate bed in 49% of patients with BCR after prostatectomy (12). The recurrences in the prostate bed were not completely covered in about 46% of cases by standard consensus target volumes (29). We found a survival benefit in the group with PET-negative disease after PET restaging, but with the adverse feature of a PSA level of 0.5 ng/mL or greater. This indicates a high curative potential of SRT in this group of patients with a lower risk of subclinical regional or distant disease. Others have also reported on the high effectiveness of SRT in PET-negative disease (30).

After SRT in patients with PET-positive lymph nodes, the incidence of new lesions found during molecular imaging within the follow-up time of the studies (12–47 mo) was 20.4% in an overview by Yang et al. (31). The OLIGOPELVIS trial, which included patients with choline PET/CT-positive lymph nodes undergoing SRT to the pelvic lymph nodes with an integrated boost to PET-positive lymph nodes, also found a low median progression-free survival time (3.8 y) after pelvic SRT and short-term androgen-deprivation therapy (32). Furthermore, the PEACE-V randomized phase 2 trial enrolled patients with BCR after prostatectomy or definitive radiochemotherapy and up to 5 pelvic lymph node metastases (detected by PSMA PET/CT in 81% of patients and by choline PET/CT in the remainder) and compared stereotactic body radiotherapy to PET-positive pelvic lymph nodes with elective nodal radiotherapy, including an integrated boost to PET-positive lymph nodes, combined with short-term androgen-deprivation therapy for 6 mo (33). The 4-y metastasis-free survival rate was 63% in the metastasis-directed stereotactic body radiotherapy group and 76% in the elective nodal radiotherapy group (*P* = 0.063). The aforementioned data show that PSMA PET–positive pelvic lymph nodes at the time of BCR indicate subclinical widespread disease and worse prognosis and that the effect of SRT on OS in this clinical situation remains unproven (as found in this study). Accordingly, the survival of patients with miT/N+ M0 disease was significantly worse than those with miT0N0M0 disease in our study.

Our study had some limitations. Because of the specific design of the PROMISE registry, details regarding the dose, volume, and duration of SRT, as well as the time-point of androgen-deprivation or androgen receptor–directed therapy, are not available. Furthermore, two thirds of patients with BCR were excluded because of missing management information. On the other hand, national guidelines were available for SRT and endocrine therapies during the period in which patients were treated, limiting heterogeneity from institution to institution. In addition, these data demonstrate the impact of SRT, given the heterogeneities occurring in the real world. However, the early introduction of PSMA PET by the participating institutions, the large sample size, and the endpoint of OS are strengths of the study. All patients underwent restaging on the basis of PSA level and anatomic and molecular imaging. Because the comparison between patients with or without SRT was not randomized, treatment selection bias related to comorbidities cannot be excluded as a potential influence on outcomes, independent of patient or physician preference. However, the smaller effect observed in the PET-locoregional disease group argues against any substantial impact of such selection bias.

## CONCLUSION

The results of this study support the benefit of SRT, particularly if no tumor is detected on PET at BCR, as reflected by a PSA level of at least 0.5 ng/mL. These findings support current clinical practice, which does not withhold SRT from patients experiencing BCR despite negative PET results.

## DISCLOSURE

This research was partly funded by Deutsche Forschungsgemeinschaft (grants FE1573/7-1/714209 and RA 3222/5-1/714210, project number 564651276), a Prostate Cancer Foundation TACTICAL Award (22TACT01), Thera4Care by the Innovative Health Initiative Joint Undertaking (grant 101172788), ILLUMINATE by the Innovative Health Initiative Joint Undertaking (grant 101172722), Novartis (2501730), AstraZeneca (95663957), and Amgen. The funders of the study had no role in study design, data collection, data analysis, data interpretation, or writing of the manuscript. Madeleine Karpinski reports stock ownership from Bayer, outside of the submitted work. Caner Civan obtained a postdoctoral fellowship from Humboldt Foundation. Stephan Beintner-Skawran reports participation on the advisory board for Novartis in Switzerland, outside of the submitted work. Karolien Goffin reports consulting fees from Novartis, Bayer, MSD, Telix Pharmaceuticals, and PSI CRO (all paid to the institution) and payment or honoraria from Telix Pharmaceuticals, Bayer, and Novartis (all paid to the institution, all outside of the submitted work. Helen Scholtissek reports a research grant from the medical faculty of University of Augsburg, outside of the submitted work. Sazan Rasul reports payment or honoraria from Janssen Pharmaceuticals, support from Telix Pharmaceuticals and Curium Pharma, and participation on the advisory board for Telix Pharmaceuticals, all outside of the submitted work. Daniele Pizzuto reports grants from Fondazione Umberto Veronesi ETS and a postdoctoral fellowship (2023–2024) and honoraria for an invited talk (Hybrid PET/CT PET/MR Hands-on Course) from University Hospital Zurich-FAPI Clinical, outside of the submitted work. Jonathan Miksch reports stock ownership in Bayer AG, outside of the submitted work. Anders Bjartell reports consulting, advisory board, or speaker′s fees from Accord, Astellas, AstraZeneca, Bayer, IPSEN, Johnson & Johnson, Pfizer, Sandoz, and Telix Pharmaceuticals; reports research grants to the institution from Astellas, AstraZeneca, Bayer, Johnson & Johnson, Movember, Roche, and Spectracure; is cofounder and a board member of Glactone Pharma AB; and is a shareholder of Glactone Pharma AB, LIDDS AB, and WntResearch AB, all outside of the submitted work. Laura Evangelista reports consulting fees for Novartis, Ergea, and Menarini, outside of the submitted work. Kambiz Rahbar reports consulting fees from Novartis, Bayer Healthcare, UroTrials, and Pharmtrace; honoraria or payment from Novartis, Bayer Healthcare, AstraZeneca, and ABX; and travel support from Bayer Healthcare and ABX GmbH, outside of the submitted work. Isabel Rauscher reports fees from GE HealthCare (speaker) and support for travel from Blue Earth Diagnostics Ltd., outside of the submitted work. Matthias Eiber reports fees from Blue Earth Diagnostics Ltd. (consultant, research funding), Novartis/AAA (consultant, speaker), Telix (consultant), Bayer (consultant, research funding), RayzeBio (consultant), POINT Biopharma (consultant), Eckert-Ziegler (speaker), Urotrials (speaker), and Janssen Pharmaceuticals (consultant, speakers bureau), Parexel (image review), and Bioclinica (image review), outside of the submitted work, and a patent application for rhPSMA. He and other inventors are entitled to royalties on sales of POSLUMA. He serves on advisory boards or steering committees for Novartis, Blue Earth Diagnostics Ltd., and Telix, all outside of the submitted work. Ken Herrmann reports grants or contracts from Novartis and SOFIE Biosciences; consulting fees from Advanced Accelerator Applications, a Novartis co., Amgen, AstraZeneca, Bain Capital, Bayer, Boston Scientific, Convergent, Curium, Debiopharm, EcoR1, Fusion, GE HealthCare, Immedica, Isotopen Technologien München, Janssen, Merck, Molecular Partners, NVision, POINT Biopharma, Pfizer, Radiopharm Theranostics, Rhine Pharma, Siemens Healthineers, SOFIE Biosciences, Telix, Theragnostics, and Y-mAbs; payment or honoraria for educational events from PeerVoice; support for American Society of Clinical Oncology participation from Janssen; participation on a data safety monitoring board or advisory board for Fusion and GE HealthCare; and ownership of stocks in SOFIE Biosciences, Pharma15, NVision, Convergent, Aktis Oncology, and AdvanCell, outside of the submitted work. Matthias Miederer reports grants or contracts from BMFTR and TheraOp; consulting fees from Novartis, Roche, and Veraxa; and payment or honoraria from Telix Pharmaceuticals and Novartis, all outside of the submitted work. Sebastian Hoberück reports work for Novartis, outside of the submitted work. Adrien Holzgreve is funded by the Deutsche Forschungsgemeinschaft (545058105); reports a contract for a research project and consulting fees from ABX; and is former chair of Young DGN (part of the German Society of Nuclear Medicine) and a member of the steering committee in Young Oncologists United as part of the German Cancer Society, all outside of the submitted work. Matteo Bauckneht reports fees from Telix Pharmaceuticals (consultant), Novartis (speaker, consultant), Johnson & Johnson (speaker), and Bayer (consultant), outside of the submitted work. Boris Hadaschik reports consulting fees from Johnson & Johnson, Bayer, ABX, Astellas, Merck, Amgen, MSD/Pfizer, Novartis, BMS, Monrol, Onkowissen, POINT Biopharma, Ipsen, AstraZeneca, Lightpoint Medical, Telix Pharmaceuticals, and Accord Healthcare; travel support from AstraZeneca, BMS, Janssen, Bayer, and Ipsen; grants or contracts from Janssen, Deutsche Forschungsgesellschaft, Novartis, and BMS; and participation on data safety monitoring boards for Johnson & Johnson and ABX. Wolfgang Fendler reports fees from SOFIE Biosciences (research funding), Janssen (consultant, speaker), Perceptive (consultant, image review), Bayer (consultant, speaker, research funding), Novartis (speaker, consultant), Telix Pharmaceuticals (speaker), GE HealthCare (speaker, consultant), Eczacıbaşı Monrol (speaker), ABX (speaker), Amgen (speaker), Urotrials (speaker), and Lilly (consultant), outside of the submitted work. Marcin Miszczyk reports support by the European Urological Scholarship Programme Scholarship of the European Association of Urology. No other potential conflict of interest relevant to this article was reported.

## Notes

KEY POINTS **QUESTION:** Does SRT improve OS in patients with prostate cancer who develop BCR? **PERTINENT FINDINGS:** The results of this study support the benefit of SRT, particularly if no tumor is detected on PET at BCR, as reflected by a PSA level of at least 0.5 ng/mL. These findings support current clinical practice, which does not withhold SRT from patients experiencing BCR despite negative PET results. **IMPLICATIONS FOR PATIENT CARE:** SRT improves OS for patients with BCR, especially those with a negative PSMA PET result.
